# Fecundity of First-Generation Captively Reared *Heterelmis comalensis* (Coleoptera: Elmidae)

**DOI:** 10.1093/jisesa/ieac024

**Published:** 2022-05-08

**Authors:** Ely Kosnicki

**Affiliations:** BIO-WEST, Inc., 1405 United Drive, Suite 111, San Marcos, TX 78666, USA

**Keywords:** riffle beetle, insect husbandry, refuge production

## Abstract

The Comal Springs riffle beetle *Heterelmis comalensis* Bosse, Tuff, and Brown is listed as endangered by the U.S. Fish and Wildlife Service. Rearing this species in captivity is important to maintaining a refuge which is a goal of the Edwards Aquifer Habitat Conservation Plan. Although captive larval production has been observed for years, the production of larvae per female has been relatively unknown. Production chambers were constructed from PVC to house one female and male and the production of larvae was monitored ca. monthly until the female died. Females were found to be iteroparous and produced 29.3 ± 37.1 larvae. The number of larvae produced was found to be a function of female longevity rather than size. Other aspects of captive production and observations from the study are discussed.

The Comal Springs riffle beetle, *Heterelmis comalensis* Bosse, Tuff, and Brown, is an aquatic beetle known from springs at Comal Springs and San Marcos Springs, in Comal and Hays Counties, Texas, respectively (Bosse et al. 1988; Gibson et al. 2008). Critical habitat designated for this species includes 15.56 ha of surface area at Comal Springs and San Marcos Springs, collectively (USFWS 1997 and 2013). All known locations of this species are found within the Edwards Aquifer, the primary water source for municipalities and agriculture around and including the city of San Antonio (Edwards Aquifer Authority 2019. https://www.edwardsaquifer.org/science-and-maps/about-the-edwards-aquifer, accessed 13 June 2019).

*Heterelmis comalensis* faces many threats to its ecosystem, including but not limited to over pumping of the aquifer, pollution, and adverse effects due to invasive species (Bowles and Arsuffi 1993). Having a functional refuge is a requirement of the U.S. Fish and Wildlife Service (USFWS) (1996) and maintaining a captive-propagating population is a goal of the Edwards Aquifer Habitat Conservation Plan. Wild collections have produced ca. 10 larvae per adult (including males) each month (Huston and Gibson 2015) in captivity at the USFWS San Marcos Aquatic Resources Center (SMARC) since 1996 (Fries 2003); however, little is known about fecundity of captively reared females. Recently, a technique was developed to separate live female and male adults, by viewing internal structures that can be seen with lateral lighting (Kosnicki 2019). The objective of this study was to count the number of larvae produced by first-generation (F1) captively reared females.

## Methods

First-generation adult *H. comalensis* were raised at the SMARC from refuge stock and from a series of rearing experiments. Acquired adults were separated by sex using a lighting technique (Kosnicki 2019). Twenty-four females were measured lengthwise from the posterior tip of the scutellum to the posterior mediad of the elytra. One female and male were placed into a production chamber. The housing of the production chambers was constructed from PVC and consisted of female 2.54-cm slip/threaded couplings fitted over each end of 8 cm long pipe and welded. Caps were made for each end by fitting a 2.54-cm slip × 1.27-cm threaded bushing into a 2.54-cm male threaded female slip coupling with a 255-µm plastic mesh placed in between and welded to form a seal. A 1.27-cm threaded spigot was screwed into the bushing with plumber’s tape to create a seal. The threaded couplings of the caps were then screwed into each end of the housing to create the flow-through tube. A hose with fresh well water supplied from the Edwards Aquifer was attached to one end of the flow-through tube spigots and flow was controlled by a ball valve (see BIO-WEST 2021).

Each production chamber contained biofilm-conditioned leaf and wood resources that beetles use for food and habitat. Biofilm-conditioned leaves consisted of dried *Platanus occidentalis* L. (Proteales: Platanaceae) leaves that were placed in flow-through containers at the SMARC and submerged in flowing Edwards Aquifer well water. After biofilms developed on the surface of the leaves for 2 to 4 mo, the leaves were considered conditioned as a resource for *H. comalensis* subjects. Biofilm-conditioned wood consisted of 1-cm diameter poplar dowels cut into lengths of 6 cm that were submerged in flow-through containers of flowing Edwards Aquifer well water for at least 3 mo but not longer than 12 mo before use.

Production chambers were inspected ca. once per month to see if the adults were still alive and to count and remove recently hatched larvae. The contents of the tube, including water, resources, and adults, were replaced back into the tube so that additional larvae could be produced and counted at a later time. When the female was alive and the male found dead during an inspection, another male (when available) was used to replace him and fecundity observations were continued for that female. Females were tracked for total fecundity until dead. The total number of larvae produced was regressed over female size (elytra length) and female longevity to determine if those relationships existed. In this way, F1 female fecundity was determined in terms of captive second generation (F2) live larvae produced.

## Results

**Fig. 1.**
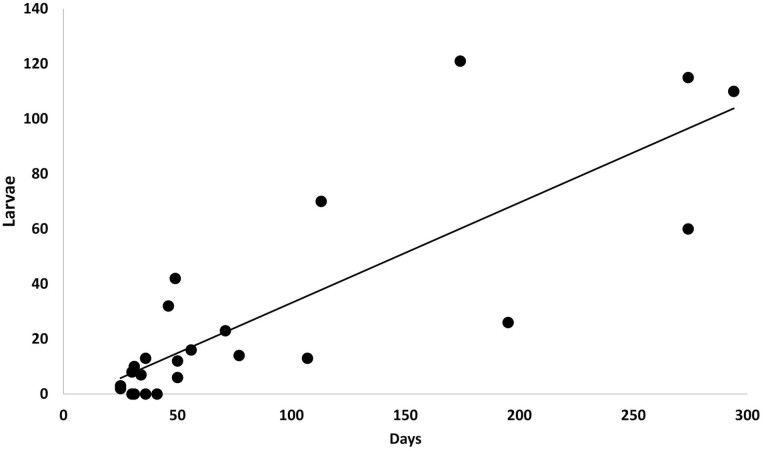
Relationship between number of larvae produced based versus number of days the female live d.

From the 24 females tracked until death, 703 larvae were produced (mean = 29.3; sd = 37.1), ranging from 0 to 121 larvae per female (Table 1). Some adults died during the first month of being placed into their respective production chambers; however, some females survived and continued to produce for almost a year. Female size was not found to be related to number of larvae produced (*F* = 1.031; df = 22; *P =* 0.314); however, the longevity of the female as number of days over all inspections was found to be related to number of larvae produced (*F* = 47.870; df = 22; *P* < 0.001; *R*^2^*=* 0.685) with a residual standard error of 21.75 (Fig. 1). The number of larvae produced per female (*L*) can be estimated as a function of days the female lives with access to a mate (*day*): *L* = 0.37 *day* – 3.33.

**Table 1.** Twenty-four first-generation captively reared *Heterelmis comalensis* females monitored for larval production

Female	Larvae produced	Days	No. checks	Female length (mm)
T.8.1	121	174	4	1.36
T.7.1	115	274	6	1.34
T.16.1	110	294	6	1.41
R.3	70	113	4	1.27
T.16.2	60	274	3	1.32
T.4.2	42	49	2	1.40
T.4. 1	32	46	2	1.29
T.15.1	26	195	4	1.26
R.2	23	71	3	1.27
T.8.4	16	56	2	1.22
T.18.1	14	77	1	1.33
A.1	13	107	3	1.34
T.10.2	13	36	1	1.44
R.1	12	50	2	1.26
T.8.2	10	31	1	1.33
T.11.1	8	30	1	1.32
T.15.2	7	34	1	1.40
T.4.3	6	50	2	1.40
T.14.2	3	25	1	1.26
T .14.1	2	25	1	1.25
T.3.1	0	41	2	1.19
T.10.1	0	36	1	1.38
T.8.3	0	31	1	1.36
T.12.1	0	30	1	1.33

Females were assigned a tracking number based on the origin of their rearing. The number of larvae produced per individual and approximate number of days that female was alive as an adult in captivity are given. The number of times the female production chamber was checked and the length of the elytra of each female is also given.

## Discussion

Results from the fecundity study clearly indicate that females are iteroparous. Even more, observations of individual females indicated that females do not produce larvae in the absence of a male. In several instances, a female that had produced larvae with a male did not produce larvae after her mate died, but began producing larvae after another male was added to her production chamber. Results from this study likely underestimate captive F1 female fecundity; it should be noted that there was a learning curve with regard to properly packing resources into production chambers and that some of the first trials of adult pairs died within the first month, likely due to poor flow conditions. Furthermore, routine checks likely increased stress and the chance of damage through handling. Therefore, some females would have probably lived longer and produced more larvae than results suggest if placed within more suitable habitats and handled less often. However, this was clearly not the case for some of the female subjects during the later portion of this experiment and it is likely that a number of these died early, possibly due to inefficient nutrition or excessive stress experienced during larvae development. Mays et al. (2021) showed that the gut microbiomes of captive *H. comalensis* had different compositions compared to wild caught ones and indicated that captive beetles may feed on bacterial communities not found in their natural habitat.

Even though female-fecundity numbers of ca. eight larvae per female per month are probably underestimated, they are higher than reported from other studies. Fries (2003) reported some of the earliest production from wild stock, recoding 38 larvae produced in 8 mo from 43 adults and Huston and Gibson (2015) reported ca. five larvae per female per month (assuming 50/50 sex ratio). Information from this study can be used to estimate how many females with access to mates in desirable conditions are needed to maintain a captive colony. A colony consisting of 10 females surviving 60 d with unlimited access to mates would produce ca. 188 larvae. Conservatively, using a 12% survival rate (based on half the rate observed from rearing experiments, BIO-WEST 2021), ca. 22 larvae would be expected to become adults. With a 50/50 sex ratio demonstrated from a rearing study (BIO-WEST 2021), 11 would be expected to be females. If F2 females have the same fecundity and survivorship as F1 females, a perpetual captive colony could theoretically be maintained; however, the genetic diversity may not reflect wild populations
